# 2015版WHO肺癌组织学分类变化及其临床意义

**DOI:** 10.3779/j.issn.1009-3419.2016.06.06

**Published:** 2016-06-20

**Authors:** 欣 杨, 冬梅 林

**Affiliations:** 100142 北京，北京大学肿瘤医院病理科 Department of Pathology, Beijing Cancer Hospital, Peking University, Beijing 100142, China

**Keywords:** WHO分类, 肺肿瘤, 组织学分级, WHO classification, Lung neoplasms, Histologic grade

## Abstract

由于过去十几年间对肺癌认识的巨大发展，尤其是在肿瘤内科学、分子生物学和放射学等领域的发展，迫切需要一个不仅病理学而是整合多学科研究成果的肺癌新分类。因此，刚刚出版的2015世界卫生组织（World Health Organization, WHO）肺、胸膜、胸腺和心脏肿瘤分类与2004 WHO分类相比有很多重要变化。新版分类在促进学科领域发展、影响学科研究、优化患者治疗和辅助评估预后方面都有显著提升。文章简要介绍重要变化如下：①2011国际肺癌研究协会/美国胸科学会/欧洲呼吸学会（International Association for the Study of Lung Cancer/ American Thoracic Society/European Respiratory Society, IASLC/ATS/ERS）分类推荐的腺癌分类主要变化；②将鳞状细胞癌重新分类为角化型、非角化型和基底样亚型，非角化型需免疫组化证明有鳞状分化；③严格限定大细胞癌的诊断只能在切除标本中做出，并对其进行瘦身，原大细胞癌分类中仅保留缺乏任何明确形态和免疫组化分化的部分；④将神经内分泌肿瘤划分到同一分类下；以及⑤目前对肺癌组织学分级的观点。

## Abstract (en)

Due in part to remarkable advances over the past decade in our understanding of lung cancer, particularly in area of medical oncology, molecular biology, and radiology, there is a pressing need for a revised classification, based not on pathology alone, but rather on an integrated multidisciplinary approach to classification of lung cancer. The 2015 World Health Organization (WHO) Classification of Tumors of the Lung, Pleura, Thymus and Heart has just been published with numerous important changes from the 2004 WHO classification. The revised classification has been greatly improved in helping advance the field, increasing the impact of research, improving patient care and assisting in predicting outcome. The most significant changes will be summarized in this paper as follows: (1) main changes of lung adenocarcinoma as proposed by the 2011 International Association for the Study of Lung Cancer/American Thoracic Society/European Respiratory Society (IASLC/ATS/ERS) classification, (2) reclassifying squamous cell carcinomas into keratinizing, nonkeratinizing, and basaloid subtypes with the nonkeratinizing tumors requiring immunohistochemistry proof of squamous differentiation, (3) restricting the diagnosis of large cell carcinoma only to resected tumors that lack any clear morphologic or immunohistochemical differentiation with reclassification of the remaining former large cell carcinoma subtypes into different categories, (4) grouping of neuroendocrine tumors together in one category, (5) and the current viewpoint of histologic grading of lung cancer.

肺癌（lung cancer, LC）的组织学分类仍是目前治疗的重要参考。世界卫生组织（World Health Organization, WHO）肺癌分类及诊断标准自40年前问世以来做出了巨大贡献。其中，1960年代第一版中将肺癌分为小细胞肺癌（small cell lung carcinoma, SCLC）和非小细胞肺癌（non-small cell lung cancer, NSCLC）是肺癌组织学分类的第一个里程碑；1980年代第二版提出了腺泡状腺癌、乳头状腺癌、细支气管肺泡癌、实性腺癌四种基本分类；2004年第四版除包括肿瘤组织学分类外，还增加了肿瘤遗传学相关信息，以肺腺癌表皮生长因子受体（epidermal growth factor receptor, *EGFR*）突变的发现最具划时代意义。

从肺癌的分类历史看，呈现逐渐从粗放向精细演进的趋势。总体而言，除2004版纳入有限的肿瘤遗传学及临床信息外，基本是局限于病理学单学科的传统组织学分类，并没有多学科的融汇贯通。直到2004版，病理学分类对治疗和预后几乎都没有有效指导意义。因为在相当长一段时间内，NSCLC被视作一个整体，临床医生只要依据这一粗放分类就可以进行无差别的治疗，特别是在活检标本诊断中，未对更具体的组织学分类（腺癌和鳞状细胞癌）予以重视，所以直到2004版分类，免疫组化及特殊染色在区分腺、鳞癌中的作用亦未被强调，甚至国际分类推荐可以使用非小细胞肺癌-非特殊类型（non-small cell lung cancer-not otherwise specified, NSCLC-NOS）的诊断术语。

2003年以来肺癌领域从基础研究到临床诊治均发生了较大变化，如薄层低剂量螺旋计算机断层扫描（computed tomography, CT）用于肺癌普查，发现了更多更早的肺癌，如EGFR-酪氨酸激酶抑制剂（EGFR -tyrosine kinase inhibitor, EGFR-TKI）为代表的靶向治疗的发展、外科微创手术的发展、放疗科立体定向放射治疗（stereotactic body radiation therapy, SBRT）治疗早期周围型肺癌的应用等，均迫切需要多学科参与新的肺癌分类，以更好的指导和服务于临床实践。

因此，在此背景上应运而生的2015年最新版WHO肺肿瘤分类，与2004版相比，发生了较大的变化。大致可归为两方面：一是最新版WHO肺肿瘤分类是第一次整合了肿瘤学、分子生物学、病理学、放射学和外科学等各个领域肺癌研究成果的多学科分类体系，多学科参与分类标准的制定，使得病理学分类能够更好的服务于临床实践及临床/基础研究，病理诊断成为患者个体化治疗的基础环节。具体体现在诊断过程强调免疫组化的应用，减少非小细胞癌作为最终诊断出现；对于晚期肺癌患者，强调组织学诊断与分子分型同样重要；第一次单独提出活检标本和细胞学标本的诊断标准。二是具体的肿瘤分类和诊断标准变化，其中变化最大的是腺癌的分类及诊断标准，除做了极小改变之外，2015版WHO肺肿瘤分类^[1]^中腺癌的分类及诊断标准几乎完全根据2011肺腺癌国际肺癌研究协会/美国胸科学会/欧洲呼吸学会（International Association for the Study of Lung Cancer/American Thoracic Society/European Respiratory Society, IASLC/ATS/ERS）国际多学科分类^[2]^执行；此外，最新版WHO分类还对鳞状细胞癌（squamous cell carcinoma, SCC）、大细胞癌（large cell carcinoma, LCC）和某些类型的神经内分泌肿瘤（neuroendocrine tumours, NET）等做了相应调整。

## 1 腺癌分类的主要变化

由于2004版WHO分类标准在预后指导方面的局限性，全世界众多病理学家一直致力于通过细化对肺腺癌各种组织学特征定性或定量评估，以提出更准确组织学预后分组的研究。许多形态特征已被证明对肺腺癌有重要的预后意义，如附壁样（lepidic）、实性（solid）和微乳头状（micropapillary）生长方式，细胞核形态、肿瘤性坏死等。特别是附壁样和微乳头生长方式作为影响肺腺癌预后的重要形态特征，在2011肺腺癌IASLC/ATS/ERS多学科新分类中，被赋予了重要地位。

### 1.1 不再使用“细支气管肺泡癌”（bronchioloalveolar carcinoma, BAC）这一术语

作为肺腺癌的一种特殊类型，1999版和2004版WHO分类标准将BAC严格定义为：不存在间质、血管或胸膜侵犯，只有肿瘤细胞呈现独特的沿支气管肺泡生长特性的腺癌亚型。BAC生物学特性以沿肺泡壁肺内扩散为主，少有肺外转移，预后明显好于其他类型肺腺癌。但在具体临床实践中，由于诊断标准把握的主观性，BAC这一术语被用于一系列肿瘤中，包括①孤立的、非浸润性、5年生存率100%的周围型小肺癌，②5年生存率接近100%的微小浸润性腺癌，③浸润性腺癌的混合亚型，④原来的粘液型和非粘液型BAC，以及⑤和生存率非常低的广泛播散性病变等。“BAC”混乱使用于这些从低度到高度恶性肿瘤中的后果是，给临床诊治和研究造成很大困扰，且给癌症登记流行病学研究带来困难。因此，2015版WHO肺腺癌分类中取消了“BAC”这一诊断术语，并针对“BAC”样生长方式，即新版分类中的附壁样生长方式这种形态特征进行细化分类，引入了原位腺癌（adenocarcinoma *in situ*, AIS）、微小浸润腺癌（minimally invasive adenocarcinoma, MIA）的概念，以及浸润性腺癌中附壁样结构的描述。

### 1.2 提出AIS的概念

与非典型腺瘤样增生（atypical adenomatous hyperplasia, AAH）一同被归入浸润前病变。2015版WHO分类标准将AIS定义为肿瘤细胞完全沿原有肺泡结构生长（纯附壁生长），无间质、血管或胸膜浸润，直径≤3 cm的局限性小腺癌。没有诸如腺泡状、乳头状、实性或微乳头状等的浸润性结构，也没有肺泡腔内肿瘤细胞存在。AIS绝大多数为非粘液性，粘液性AIS极少见。AIS完全切除后预后极好，5年无病生存率达100%。组织学上，AIS无真正浸润证据，故新版WHO分类将AIS归入浸润前病变，与AAH一起分别对应鳞状细胞原位癌和鳞状上皮不典型增生，由此成为肺实质切除范围可以缩小的理论依据。

### 1.3 引入MIA的概念作为独立分类

定义为以附壁样结构为主伴有最大径≤5 mm浸润灶的孤立性小腺癌（≤3 cm）。多个浸润灶以直径最大者为准，而非将多个浸润灶直径相加。MIA如果完全切除预后也非常好，5年无病生存率及无复发生存率100%。

### 1.4 在浸润性腺癌中取消2004版WHO腺癌分类中腺癌混合亚型这一分型

提倡全面、详细的组织学诊断模式，根据肿瘤主要的生长方式对浸润性腺癌进行分类。以5%增量对附壁样（lepidic）、腺泡样（acinar）、乳头状（papillary）、实性（solid）及微乳头样（micropapillary）5种不同生长方式所占比例进行半定量评估，达到5%即在诊断中进行描述。

根据2004版WHO肺肿瘤分类及诊断标准，超过90%的腺癌都被归为混合亚型，而随访发现相同分期的混合亚型患者间预后差别也很大，说明2004版WHO肺腺癌组织学分类预后意义有限。

由于新版WHO肺肿瘤分类中腺癌的分类几乎完全脱胎于2011肺腺癌IASLC/ATS/ERS多学科新分类标准，所以自2011腺癌新分类标准提出以来，已有大量在切除肺腺癌标本中验证新版腺癌分类中提倡的主要生长方式预后价值的研究涌现，结果显示改良的分类标准根据主要成分生长方式对浸润性腺癌的分层，能够很好地揭示患者临床转归，具有一定预后意义。多数结论显示，附壁样结构为主型肿瘤预后最好，微乳头及实性结构为主型肿瘤预后不佳，腺泡状及乳头结构为主型腺癌预后介于其间。有限度切除的临床试验可根据此类预后亚型对患者进行分层，因为与附壁样结构为主型肿瘤相比，微乳头和/或实性结构为主亚型患者可能更适于接受全肺叶切除。

### 1.5 引入浸润性粘液腺癌（invasive mucinous adenocarcinoma）概念

取代原2004版WHO分类中不符合AIS和MIA诊断标准的那部分粘液型BAC（符合AIS和MIA诊断标准的则分别定义为粘液型AIS和粘液型MIA）。诸多研究表明，在临床、影像学、病理学及基因方面，之前划分的粘液型BAC与非粘液型BAC相比均有着较大不同。浸润性粘液腺癌常缺TTF-1表达，CT上常见合并结节及空气支气管征，存在多中心、多叶性及双侧肺侵犯倾向，预后不良。肺腺癌尚无特定的组织学-分子事件关联，这与肉瘤及淋巴瘤不同，而浸润性粘液型腺癌中存在高比例*KRAS*突变而少见*EGFR*突变，则为最强的组织学-分子事件关联。

### 1.6 对浸润性腺癌变异型的调整

① 取消2004版WHO分类中的透明细胞和印戒细胞腺癌亚型，视其为一种细胞学特征。原因是，二者作为细胞学变化，多种组织学类型的腺癌都会出现；尽管如此，一旦存在此类特征，即便其数量再少也应记录，尤其是在鉴别肺内多灶腺癌为原发或转移时，其存在可能有一定鉴别意义。②取消2004版WHO分类中粘液性囊腺癌，认为这是胶样型腺癌的局部形态学表现。③新增肠型腺癌这一亚型，但要与消化道来源的腺癌进行鉴别。由于肺原发肠型腺癌亚型形态学上以类似结直肠癌为特征，而且部分肿瘤具有肠型分化，如COX2和CK20阳性，而CK7阴性，所以目前从形态及免疫表型上与转移性结直肠癌鉴别困难。只有在临床完全除外了存在原发肠癌之后才能认为是肺原发肠型腺癌。

## 2 鳞状细胞癌的调整

这里主要讨论针对手术切除标本的调整。2004版WHO分类中鳞状细胞癌主要包括乳头状、透明细胞、小细胞和基底样亚型。但是，这个分型并没有太多意义，因为乳头、透明细胞和小细胞亚型非常罕见。而且回顾研究发现，鳞状细胞癌小细胞亚型这一术语可能不是一个很好的选择，因为应用于临床，可能与小细胞癌相混淆，所以在2015版WHO分类中，已将这个亚型删除；与腺癌中的情况相似，透明细胞变目前被认为是一种细胞学特征，在角化型或非角化型鳞状细胞癌中均发生，所以2015版WHO分类中，亦不再将透明细胞亚型视为一个正式分类，但可在诊断中被描述成“伴透明细胞特征”，同时要描述其含量，即使其所占比例很小；另外，随着对原分类中大细胞癌亚型中的基底样癌实际上可表达鳞状分化免疫标记物的认识，基底样癌从大细胞癌分类中分离出来，变成了鳞状细胞癌的基底样亚型。基于以上原因，鳞状细胞癌分类被修改为包括角化、非角化和基底样亚型三个类型，相似于头颈部肿瘤WHO分类中鼻咽癌的分类。区分角化型和非角化型鳞状细胞癌似乎并没有预后意义。有些研究提示基底样型鳞状细胞癌预后差，但是结论尚存争议，临床病理实践中该亚型有时主要需与小细胞癌进行鉴别。

## 3 大细胞癌的调整

首先需要强调大细胞癌的诊断只能在手术切除肿瘤做出，所以这个属于不适用于小活检和细胞学。在2004版WHO分类中，大细胞癌包括很多亚型，如大细胞神经内分泌癌、基底样癌、淋巴上皮样癌、透明细胞癌和大细胞癌伴横纹肌表型。但2004版WHO分类并未提倡利用腺或鳞状分化免疫标记物对这些肿瘤进行评估。然而，2015版WHO分类指出，如果TTF-1或p40阳性，显示实性生长方式的肿瘤应分别被重新归类为实性型腺癌或非角化型鳞状细胞癌。该变化基于遗传学和免疫组化的研究，说明原来被归类为大细胞癌的肿瘤是一组有腺样、鳞状分化或没有任何免疫表型和基因表型的异质性肿瘤。来自NCI流行病学监视和终末结果登记的流行病证据显示，从TTF-1被用于临床诊断起，大细胞癌的诊断开始减少，这也反映了在临床实践中病理医师开始对大细胞癌进行重新分类。所以，2015版WHO分类强调免疫组化和特殊染色在诊断中的应用后，大细胞癌的诊断将进一步减少。

此外，与2004版WHO分类相比，其他大细胞癌亚型的调整如下：将大细胞神经内分泌癌归入神经内分泌肿瘤；基底样癌归为鳞状细胞癌亚型之一；淋巴上皮样癌归入“其他和未分类癌”分组中；透明细胞和横纹肌样表型均被视为一种细胞学形态，不再作为独特的组织学亚型，因为它们可发生于腺癌或鳞状细胞癌等不同的组织学类型中。

## 4 神经内分泌肿瘤的调整

肺神经内分泌肿瘤首次被报道于1926年，由Barnard等描述为“纵膈燕麦样肉瘤”，实则为肺“燕麦细胞癌”。1937年，Hanperl等发现所谓“支气管腺瘤”与已经报道的胃肠类癌相似，故同样命名为“类癌”，此两类肿瘤于1967年列入首版WHO肺癌分类中。小细胞癌从最初的淋巴细胞样（燕麦细胞型）、多角型或梭型、其他型（包含非小细胞癌成分）三个亚型到1982版WHO分类中的燕麦细胞型、中间型、复合型，直到1999版WHO分类仅保留小细胞癌和一个亚型即复合型（与腺癌、鳞状细胞癌或大细胞癌等复合），并延续至今。20世纪90年代逐步完善了肺神经内分泌肿瘤种类的名称，即类癌（典型类癌、不典型类癌）、大细胞神经内分泌癌、小细胞癌。

2015版WHO分类对神经内分泌肿瘤做出的最大调整，应该是将大细胞神经内分泌癌从2004版WHO大细胞癌分类中分离出来，根据细胞起源，将其与类癌、不典型类癌、小细胞癌一起，统归入神经内分泌肿瘤，虽然争议巨大。除此之外，各类型的诊断标准与1999版、2004版WHO分类没有大的变化。

典型类癌和不典型类癌通常以手术切除为主要治疗手段，二者中典型类癌更为惰性，即使出现局部淋巴结转移，临床也会稳定多年，5年、10年生存率几乎达到90%。不典型类癌早期切除临床预后良好，但如果早期播散则术后复发危险性增高。5年、10年生存率为70%和35%。大细胞神经内分泌癌尽管与小细胞癌同属高级别神经内分泌癌，目前治疗仍采用早期手术切除，而对于不能切除的病例，有研究报道利用小细胞癌的化疗方案可使患者获益。由于病例数有限，尚无肯定的预后指标。大细胞神经内分泌癌5年、10年生存率约为30%和10%。在肺神经内分泌肿瘤中最具侵袭性的当属小细胞癌，通常分为局限期和广泛期，但目前也推荐使用肿瘤-淋巴结-转移（tumor-node-metastasis, TNM）分期。对于小细胞癌，除非外周性早期病灶可手术切除，大部分病例手术并不能延长生存，所以化疗或放化疗治疗为主要治疗手段，但远期疗效较差，5年生存率大约10%，10年生存率小于5%。

## 5 肺癌的组织学分级

目前，肺癌中除了大细胞癌和多形性癌通常被认为是高级别，以及传统的将典型类癌视为低级别、非典型类癌视为中级别、大细胞神经内分泌癌和小细胞癌视为高级别之外，大部分肺癌没有公认明确的分级系统。在手术标本中，有文献推荐肺腺癌根据结构或核或联合两者进行分级的方法。目前，根据腺癌中最主要的结构分级似乎是一个简单有效的方法。大多数研究显示附壁样腺癌为低级别，腺泡状和乳头状腺癌是中级别，实性和微乳头腺癌为高级别。对中级别的腺泡状和乳头状腺癌进一步分层会更好，核级别和核分裂数对进一步分层可能有帮助。但是，需要更多的研究验证哪种分级方式与临床实践更具相关性。对于手术切除的鳞状细胞癌标本而言，可获取的相关数据更少，但核直径被证实为预后较差的独立预后因子。总之，肺癌的组织学分级标准仍需要更多研究提供依据。

病理分类的本意在于期望回答肺癌的本质，然而没有任何一种分类可以囊括肺癌的所有特性，任何一版分类也都是阶段性的，需要接受时间及临床应用的评价，目前的分类也存在有待改进的地方，如判断微小浸润癌的标准等。病理分类的生命力在于指导治疗和评估预后，目前看来新版肺癌分类较前几版在这一方面有较大提升，并能与后续基于分子事件的临床试验更好的整合。而新版肺癌分类的生命周期、对肺癌精准医疗的推动，有待我们每一个在肺癌领域内耕耘的人去验证和发展。
